# Management of Postural Orthostatic Tachycardia Syndrome in the Absence of Randomized Controlled Trials

**DOI:** 10.19102/icrm.2021.120705

**Published:** 2021-07-15

**Authors:** Asim Kichloo, Michael Aljadah, Blair Grubb, Khalil Kanjwal

**Affiliations:** ^1^Department of Internal Medicine, Central Michigan University, Saginaw, MI, USA; ^2^Department of Internal Medicine, Samaritan Medical Center, Watertown, NY, USA; ^3^Department of Internal Medicine, Medical College of Wisconsin, Milwaukee, WI, USA; ^4^Section of Electrophysiology, The University of Toledo Medical Center, Toledo, OH, USA; ^5^Section of Electrophysiology, Michigan State University McLaren Greater Lansing, Lansing, MI, USA

**Keywords:** Managemen, nonpharmacological, pharmacological, postural orthostatic tachycardia syndrome

## Abstract

Postural orthostatic tachycardia syndrome (POTS) is a clinical syndrome causing patients to experience light-headedness, palpitations, tremors, and breathlessness upon assuming an upright posture. Despite the absence of available long-term, multicenter, randomized controlled trial data, this literature review aims to concisely present the nonpharmacological and pharmacological interventions that have been used in the treatment of POTS reported to date by cross-sectional studies, cohort studies, and retrospective studies. We attempt to classify treatments as first-, second-, and third-line therapies based on our own experience and available data.

## Introduction

### Prevalence of postural orthostatic tachycardia syndrome

The prevalence of postural orthostatic tachycardia syndrome (POTS) in the United States is roughly 0.2%.^1^ In a cross-sectional, online, community-based survey that contained a sample of POTS patients, the authors demonstrated that 93% of the 4,835 patients affected were white women of child-bearing age,^2^ and half of the study population had developed symptoms in adolescence by 14 years of age.^2^ Furthermore, 83% of patients reported having another comorbid condition, such as depression, migraine, irritable bowel syndrome, or chronic fatigue syndrome, which can cause episodic tachycardia symptoms in the absence of POTS.^2,3^ POTS has also been documented, albeit more rarely, in association with multiple sclerosis, after bariatric surgery (though it is difficult to rule out postoperative hypovolemia) and in concert with traumatic brain injuries.^4–6^

### Definition of postural orthostatic tachycardia syndrome

POTS is a clinical syndrome diagnosed after thorough history-taking and physical examination.^1^ Patients with POTS typically experience symptoms such as light-headedness, palpitations, tremors, blurry vision, exercise intolerance, and breathlessness after assuming an upright position.^1^ There is also an increase in the heart rate by more than 30 bpm with the absence of orthostatic hypotension, which is defined as having a greater than 20-mmHg drop in systolic blood pressure or a 10-mmHg drop in diastolic blood pressure within three minutes of assuming an upright posture from a sitting or lying position.^1^

The onset of POTS has been attributed to dysautonomia, hypovolemia, deconditioning, a hyperadrenergic physiologic state, hypervigilance (unusual focus on bodily sensations), or any combination of these conditions.^1^ In patients with dysautonomia, there is sympathetic denervation (measured by the amount of norepinephrine in the veins) in the lower extremities, leading to venous pooling and compensatory tachycardia.^1,3,7^ In patients with hypovolemic POTS, there is an inappropriate reduction in plasma renin activity and aldosterone levels despite low blood volumes.^1,3,8^ Those who are deconditioned were found to have low stroke volume, though it is unclear whether this is the cause of POTS in this patient group or a secondary finding.^1^ Lastly, in patients with hyperadrenergic POTS, there is an increase in plasma norepinephrine levels when standing, which can be due to a loss-of-function mutation causing decreased norepinephrine transporter levels, which reduces norepinephrine reuptake.^3,9,10^

### Clinical presentation and diagnostic evaluation

In the same cross-sectional, online, community-based survey of POTS patients, the most common presenting symptoms in those diagnosed with POTS included light-headedness (99%), tachycardia (97%), presyncope (94%), shortness of breath (88%), and palpitations (87%).^2^ When evaluating patients for POTS, clinical history of symptoms, precipitating events, and triggers can lead to the diagnosis.^11^ Furthermore, orthostatic vital signs can reproduce symptoms to confirm.^11^

The Canadian Cardiovascular Society recommends a 12-lead electrocardiogram and routine set of laboratory tests be performed before making a diagnosis of POTS, as other conditions such as electrolyte abnormalities, thyroid abnormalities, adrenal gland abnormalities, and blood count abnormalities can also cause orthostatic tachycardia.^11^ As such, the presence of orthostatic tachycardia alone is not sufficient to make a diagnosis of POTS, and failure to use caution when making a diagnosis of POTS can lead to unnecessary testing, unwarranted treatment, and treatment side effects. The Canadian Cardiovascular Society does not recommend routine use of ancillary cardiac testing, such as echocardiograms or loop monitoring, but these can be used at the physician’s discretion to continue ruling out other causes of orthostatic tachycardia.^11^ Once a diagnosis of POTS is made, additional testing is indicated if the symptoms are refractory to initial therapies and if therapy needs to be more targeted.^11^ Examples of additional tests include autonomic testing and blood volume testing if there is a suspicion for dysautonomic POTS or hypovolemic POTS.^11^

To date, there are few published articles that combine, condense, or review the nonpharmacological and pharmacological management options available to POTS patients. This article aims to provide a starting point in the form of a review that discusses the different treatment modalities, the known mechanisms of actions, and the indications for use for each treatment modality. With this review, the hope is to encourage long-term, multicenter, randomized controlled trials to better develop concrete treatment guidelines for POTS.

## Methods

A literature review was performed by the lead author for articles related to POTS management. We searched PubMed, Google Scholar, the Cochrane Library, and Ovid MEDLINE using the keywords: “POTS” and “management” together with “pathophysiology,” “prevalence,” “therapy,” “treatment,” “diagnosis,” or “presentation.” We then reviewed studies published in English between the years 2000 and 2020. In addition, we reviewed the guidelines of various professional organizations, including the Heart Rhythm Society and the Canadian Cardiovascular Society. After the literature search, 32 articles were chosen based on their relevance to the topic of interest; these articles were reviewed and their inclusion agreed upon by all the authors. Excluded articles consisted of duplicates, abstracts, articles not published in English, and works that were unpublished or unrelated to the topic of interest. Of note, no multicenter, long-term, randomized controlled trials were found that specifically addressed the management of POTS.

### Management

**Figure 1:**
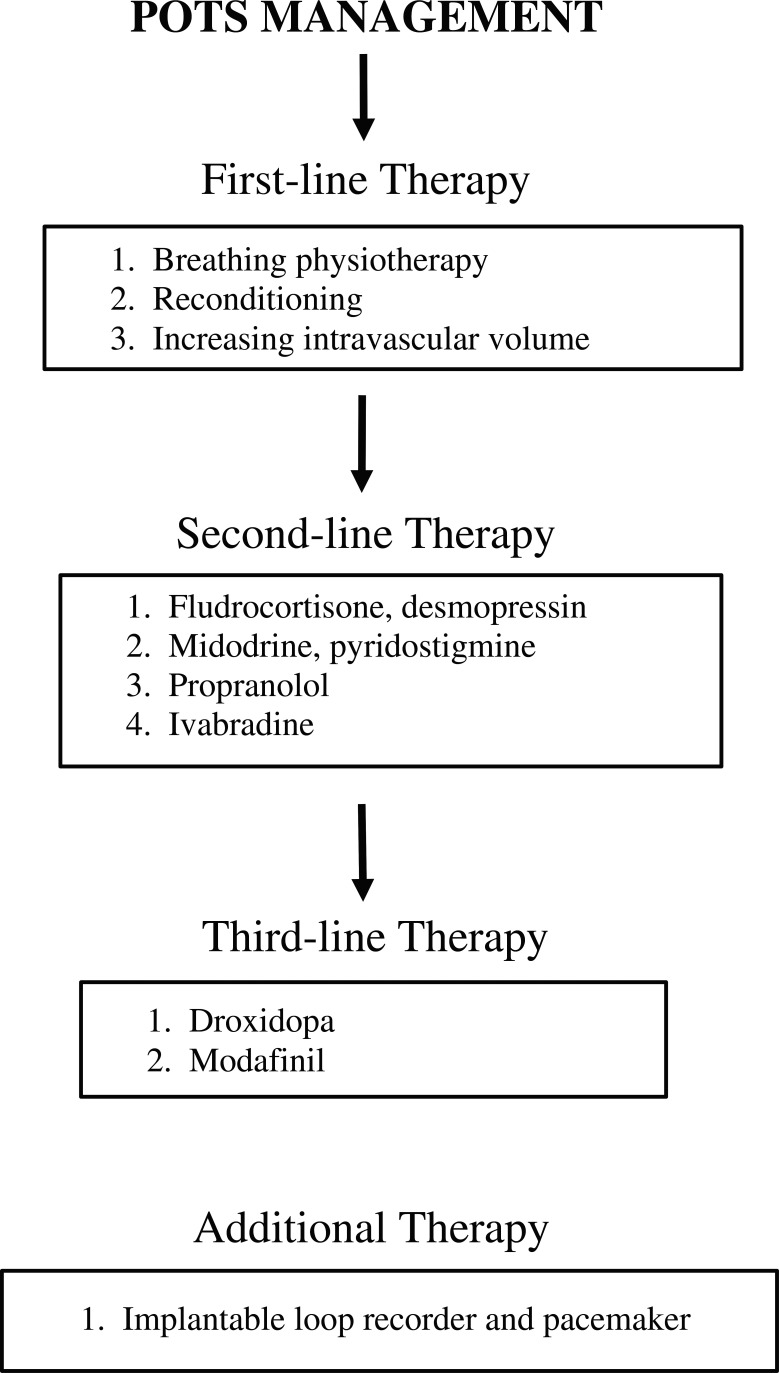
Summary of first-, second-, and third-line therapies of POTS. Treatment options are proposed in a step-wise approach based on effectiveness and side-effect profile. Nonpharmacologic therapies have the lowest side-effect profile and should be trialed first. These options are followed by second-line therapies, which have well-documented effectiveness as was described in this report. Lastly, third-line therapies are those therapies with early or little effectiveness data or with significant side effects.

The management of POTS can be divided into non-pharmacologic and pharmacologic categories. We also propose a first-, second-, and third-line therapy approach based on the effectiveness of the therapy and the risk profile of each therapy, which is summarized in **Figure 1**.

#### Nonpharmacologic management

Nonpharmacologic management options are first-line therapies.^1,11^ This includes cessation of medications that may worsen POTS, such as norepinephrine reuptake inhibitors.^1^ Many nonpharmacologic therapies revolve around symptom control.

#### Breathing physiotherapy

A common symptom of POTS patients includes the sensation of breathlessness when standing.^12^ Boulding et al. proposed that the breathlessness observed in POTS patients could be divided into five categories: hyperventilation syndrome, periodic deep sighing, thoracic-dominant breathing, forced abdominal expiration, and thoraco-abdominal asynchrony.^13^ Classifying breathlessness into these categories can help to address the root cause of symptoms through breathing physiotherapy. In a retrospective observational cohort study of 100 patients, 99 of whom were female, the authors studied the effects of breathing.^14^ The physiotherapy intervention included education on proper breathing techniques with breathing retraining exercises and frequent meetings with a specialized respiratory physiotherapist.^14^ The breathing retraining exercises involved attempting to achieve nasal breathing, normal respiratory rates, normal tidal volume, proper inspiratory/expiratory ratios, and proper thoraco-abdominal excursion, which are mechanisms that are naturally compromised in the setting of inappropriate tachycardia and during the feeling of breathlessness.^14^ Of the 66 patients who remained committed to the physiotherapy intervention, 97% reported improvements in symptom burden with improved respiratory rates and improved breath-hold times after a mean of just three sessions.^14^ However, further investigation is warranted to rule out a placebo effect as this small retrospective cohort study had no control group. However, breathing physiotherapy may be an effective option for the management of a very burdensome symptom of breathlessness in patients with POTS.

#### Reconditioning

Some of the symptoms observed in POTS overlap with exercise deconditioning. For example, low exercise tolerance, high heart rate responses during submaximal exercise, and reduced stroke volume during exercise have been observed in POTS patients.^15^ Graded-exercise training may be beneficial for symptom control in POTS.^16^ Fu et al. demonstrated an increased renin–angiotensin–aldosterone system response when standing after three months of graded-exercise training in comparison with no increased renin–angiotensin–aldosterone system response achieved with β-blockers, effectively blunting the orthostatic tachycardic response in POTS patients.^16^ Exercise conditioning may therefore be a medication-free solution that provides symptomatic relief.

#### Increasing intravascular volume

In patients with hypovolemic POTS, management is centered around maintaining adequate intravascular volume. This can be done by increasing the fluid intake to 2 to 3 L/day and increasing the salt intake to 10 to 12 g/day, even with the use of salt tablets.^1,17^ Furthermore, other measures that reduce venous pooling in the lower extremities, such as the use of compression garments or abdominal binders, can help to maintain adequate intravascular volume.^11,17^

### Pharmacologic management

If symptoms persist through nonpharmacologic treatment, pharmacologic options are available. Data for the use of these medications are largely gleaned from retrospective cross-sectional and cohort studies. There are no long-term, multicenter, randomized controlled trials for pharmacologic options for POTS.^11^ Doses of medications studied in POTS are summarized in **Table 1**.

#### Fludrocortisone and desmopressin

For hypovolemic POTS, fludrocortisone and desmopressin may help with symptom control. Fludrocortisone is a mineralocorticoid with glucocorticoid activity that works by increasing salt and water retention, which subsequently increases the intravascular volume, preventing compensatory tachycardia.^17^ Desmopressin is an antidiuretic synthetic peptide that increases the aquaporin channels in the collecting duct to also increase the intravascular volume. Fludrocortisone has a wide array of side effects, though, including hypertension, hypokalemia, and hypomagnesemia.^1^ Considering desmopressin, a single-center, randomized, crossover study of 30 patients demonstrated that patients taking desmopressin demonstrated a significantly lower standing heart rate and improved symptom burden relative to placebo therapy in patients with hypovolemic POTS.^18^ However, a major side effect of desmopressin is hyponatremia, and patients should be monitored closely for any side effects.

#### Midodrine and pyridostigmine

In dysautonomic POTS, medications are directed at increasing vascular tone to prevent blood pooling and subsequent orthostatic tachycardia. Midodrine is an α1-adrenergic receptor agonist that has specifically been shown to improve symptoms in dysautonomic POTS.^19^ In a small, double-blind, placebo-controlled crossover study of 20 patients with POTS, midodrine was shown to improve orthostatic tachycardia by increasing peripheral vascular resistance, lowering peripheral venous flow, and reducing peripheral venous capacitance relative to placebo therapy when administering doses of 2.5 to 10 mg three times daily.^19^

With regard to pyridostigmine, the effect of acetylcholinesterase inhibition to increase vascular tone in the setting of dysautonomic POTS has also been studied. In a small, placebo-controlled crossover study of 17 patients with POTS, the orthostatic heart rate was significantly improved at two and four hours after the administration of 30 mg of pyridostigmine relative to placebo therapy.^20^ Study participants reported a significant improvement in symptom burden on pyridostigmine after four hours without an observed effect on blood pressure, demonstrating that proper vascular tone in these patients plays a significant role in dysautonomic POTS.^20^

Importantly, the use of the aforementioned drugs is not without side effects. The administration of midodrine and pyridostigmine, either in combination or alone, can cause worsening of the hyperadrenergic state in patients with hyperadrenergic POTS.^19^ Pyridostigmine can also cause diarrhea.^11^

#### β-blockers

β-blockade for heart-rate control in the setting of inappropriate tachycardia has shown to be effective and to improve overall symptoms, especially with the use of propranolol.^21^ In a randomized crossover study of 54 patients with POTS who were trialed with 20 mg of propranolol versus placebo, orthostatic heart rates were significantly lower with propranolol use as compared with among those on placebo.^22^ In addition, symptom burden was improved in patients on low-dose propranolol relative to placebo therapy.^22^ Furthermore, when comparing 80-mg propranolol doses and 20-mg propranolol doses in patients with POTS, symptom control after two hours was achieved with the lower dose.^22^ While β-blockers were ineffective in reducing other known factors that may exacerbate the onset of POTS, improvements in tachycardia and symptom control may provide symptomatic relief. However, as POTS tends to be episodic in nature and propranolol is a nonspecific β-blocker, there is a risk for bradycardia and hypotension.

#### Ivabradine

Ivabradine is a selective funny current blocker that does not affect blood pressure.^23^ Due to the selective inhibition of the sinus node, ivabradine has been used in the treatment of POTS. In a retrospective study of 49 patients with POTS who received ivabradine for symptom control, 88.4% reported improvement in their palpitations and 76.1% of patients reported improvement in their light-headedness.^24^ Objectively, ivabradine treatment also decreased orthostatic heart rates, even to within normal ranges, in some patients.^24^ Ivabradine is a teratogen, and adequate contraception is required for women of child-bearing age.^11^

#### Droxidopa

Droxidopa is an amino acid that is converted to norepinephrine when orally administered.^25^ The presumption that droxidopa increases vascular tone and improves symptoms was reviewed in a retrospective study of 37 patients with POTS who were prescribed droxidopa for dizziness, fatigue, and syncope.^25^ While patients did report a decrease in the symptoms of dizziness by 16.2%, fatigue by 27.1%, and syncope by 18.9%, only 27% of patients reported improved quality of life and almost half stopped the treatment due to side effects or ineffectiveness.^25^ Unfortunately, the effects of droxidopa on orthostatic tachycardia have yet to be studied. Side effects include worsening dizziness, headaches, and hypertension.

#### Modafinil

POTS patients also have symptoms that are not orthostatic in nature, one of which is fatigue. Modafinil is a dopamine reuptake inhibitor that acts as a stimulant and has been trialed in patients diagnosed with POTS who exhibit fatigue and decreased alertness or concentration.^26^ When initially purposed as a treatment, modafinil was actually shown not to have an effect on heart rates in patients diagnosed with POTS relative to placebo patients in a randomized crossover trial.^26^ Meanwhile, in a retrospective nonrandomized analysis of 60 patients with POTS, 67% reported a significant improvement in fatigue and alertness on 100 to 200 mg of modafinil daily.^27^ Therefore, while modafinil may not objectively improve tachycardia in POTS patients, it may have a role in symptom control in patients exhibiting fatigue.

### Special considerations

#### Postural orthostatic tachycardia syndrome and neurocardiogenic syncope

Patients with POTS usually do not present with syncope. However, a subset of patients has neurocardiogenic syncope (NCS) in addition to POTS. In a retrospective study, 39 patients with POTS and NCS who had unusually frequent syncope underwent loop recorder insertion. These patients demonstrated either prolonged asystole of more than six seconds or severe bradycardia of less than 30 bpm during their syncopal episode.^28^ All 39 patients underwent dual-chamber pacemaker placement to prevent prolonged asystole and severe bradycardia and syncope were completely eliminated, suggesting that, in patients with POTS and NCS, loop recorder and subsequent pacemaker placement may have a role in significantly reducing symptoms.^28^ While pacemaker placement eliminated their frequent syncope, these patients continued to have orthostatic tachycardia. There is currently no utility of loop recorder insertion and pacemaker placement in patients with pure POTS without concurrent NCS.

## Conclusion

The treatment of POTS remains complex and, at times, difficult to execute as there are currently no long-term, multicenter randomized clinical trials to guide treatment. Once a diagnosis is made, which is largely a diagnosis made by exclusion, treatment strategies are mainly chosen to alleviate the symptoms. While nonpharmacologic options are available, including breathing intervention and graded exercise, there is a lack of established, convincing data for any pharmacologic option. Patients also have symptoms that are not orthostatic in nature and physicians need to address those as well.

## Figures and Tables

**Table 1:** Summary of Pharmacological Therapies and Their Dosages and Side Effects

Therapy	Dosage Studied	Possible Side Effects of the Studied Dosage
Fludrocortisone^17^^*†^	0.2–0.3 mg daily	Hypertension, headache, hypokalemia, lower extremity edema, and congestive heart failure exacerbation
Desmopressin^18^	0.2 mg daily	Hyponatremia, edema, and headache
Midodrine^19^^*†^	2.5–10 mg TID	Hyperadrenergic state (most commonly tremor, anxiety)
Pyridostigmine^20^^*†^	30 mg daily	Hyperadrenergic state (most commonly tremor, anxiety), diarrhea
Propranolol^22^^*†^	20 mg daily	Fatigue, bradycardia, hypotension
Ivabradine^24^^*†^	2.5–10 mg daily	Luminous phenomena, visual brightness, teratogenic
Droxidopa^25^	100–600 mg TID	Headache, dizziness, hypertension
Modafinil^27^^*^	100–200 mg daily	Headache

BID: twice daily; TID: three times daily.

^*^Therapy was discussed in the 2015 Heart Rhythm Society expert consensus statement.

^†^Therapy was discussed in the 2020 Canadian Cardiovascular Society consensus statement.
